# A Hybrid FE-ML Approach for Critical Buckling Moment Prediction in Dented Pipelines Under Complex Loadings

**DOI:** 10.3390/ma18204721

**Published:** 2025-10-15

**Authors:** Yunfei Huang, Jianrong Tang, Dong Lin, Mingnan Sun, Jie Shu, Wei Liu, Xiangqin Hou

**Affiliations:** 1 Institute of Safety, Environmental Protection, and Technical Supervision, PetroChina Southwest Oil & Gasfield Company, Chengdu 610095, China; 2 PetroChina Southwest Oil & Gasfield Company, Chengdu 610094, China; 3 School of Mechatronic Engineering, Southwest Petroleum University, Chengdu 610500, China; 4 School of Civil Engineering and Geomatics, Southwest Petroleum University, Chengdu 610500, China

**Keywords:** dents, pipelines, critical buckling moment, complex loading, finite element analysis, machine learning

## Abstract

Dents are a common geometric deformation defect in pipelines where the dented section becomes susceptible to local buckling, significantly threatening the integrity and reliability of the pipeline. This paper developed a novel finite element (FE) machine learning (ML)-based approach to analyze and predict the critical buckling moment (CBM) of dented pipelines under combined internal pressure and bending moment (BM) loading. By quantifying the parametric effects on CBM and developing a dataset, an Extreme Learning Machine (ELM) framework through hybrid algorithm integration, combining Bald Eagle Search (BES), Lévy flight, and Simulated Annealing (SA), was proposed to achieve highly accurate CBM predictions. This study offers valuable insights into evaluating the buckling resistance of dented pipelines subjected to complex loading conditions.

## 1. Introduction

Pipelines function as an essential medium for delivering fossil fuels (oil, natural gas) alongside clean energy carriers like hydrogen [1,2]. This safe, efficient, and economical mode of energy transportation holds a significant position in global energy security strategic planning [3,4]. Nevertheless, pipeline transportation is susceptible to third-party damage, corrosion, material defects, and operational interruptions, which can lead to a range of imperfections in pipelines, including geometric deformation, crack-like defects, and metal loss [5,6]. These deficiencies adversely affect the structural integrity and stability of pipelines, seriously hampering their safe operation [7]. Additionally, pipelines are typically buried, making them vulnerable to the impacts of ground movement that may cause them to undergo bending, compression, twisting, and local buckling or even fracture [8]. These deformations can result in the leakage of transport media and transportation interruptions, leading to substantial economic losses. An impressive incident is the 2018 Guizhou natural gas pipeline fracture in China due to a landslide, which caused one fatality, 23 injuries, and a direct economic loss of CNY 21.45 million [9]. Hence, an in-depth understanding of pipeline defects and their stress states is of paramount importance for enhancing the resilience and durability of energy infrastructures.

**Figure 1:**
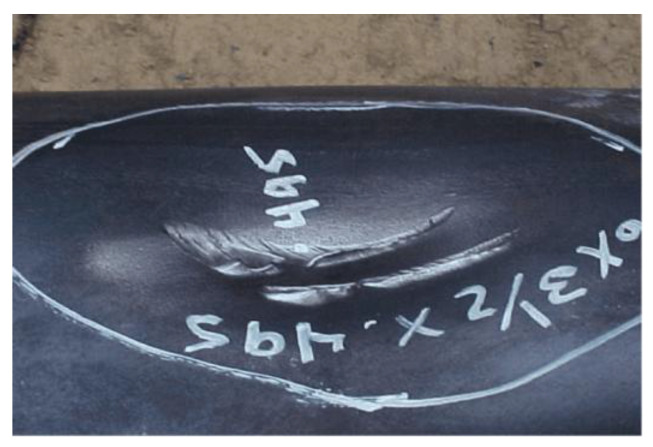
View of a pipeline dent.

Dents are the most typical geometric deformation defects on pipelines, as shown in Figure 1, usually caused by damage during pipeline manufacturing, transportation, and installation processes [10,11,12]. The presence of dents may lead to local stress concentration in the pipeline, making the stress level in that area far exceed the yield limit of the pipeline material [13,14]. This high-stress state accelerates fatigue damage and crack initiation in the pipeline. Over time, cracks may propagate to a certain extent, resulting in pipeline leakage or rupture. Dents can also affect the pipe’s in-line inspection (ILI) process. During ILI operations, dents may act as obstacles within the pipeline, causing the detector to become stuck or damaged [15]. Additionally, due to various factors such as geohazards, construction deviations, and external forces, local buckling is prone to occur at the dented pipe segment, posing a significant threat to the safe running of the pipeline [16].

Previous studies had made some achievements in the buckling behavior of pipelines. Shuai et al. [17] established a validated FE model to investigate how BM, axial force, and corrosion geometry influence the burst capacity of steel pipelines, leading to the creation of a new burst pressure prediction model for pipelines under combined BM and axial compression:

(1) $$P_{limit}=\frac{4\sigma_{u}}{{\left(\sqrt{3}\right)}^{0.239{\left(\frac{\sigma_{u}}{\sigma_{y}}-1\right)}^{0.596}+1}}\frac{t}{D}{\left(1-0.23578\frac{F}{\pi Dt\sigma_{y}}\right)}^{2}{\left(1-0.12923\frac{M}{D^{2}t\sigma_{y}}\right)}^{3.61084cos\theta -1}\;\left\{1-\frac{\delta }{t}\left(1-\left(0.1209{\left(1-{\left(\frac{W}{\pi D}\right)}^{2}\right)}^{4}+0.8791exp\left(\frac{-1.1556L}{\sqrt{Dt}}\right)\right){\left(1-\frac{\delta }{t}\right)}^{-0.08192}\right)\right\}$$

where $P_{limit}$, $\sigma_{u}$, $\sigma_{y}$, t, D, F, M, δ, W, and L denote the burst pressure [MPa], ultimate tensile strength [MPa], yield strength [MPa], the pipe’s wall thickness [mm], the pipe’s outer diameter [mm], axial force [MPa], bending moment [MPa], corrosion depth [mm], corrosion width [MPa], and corrosion length [MPa], respectively. In a similar study from Mondal and Dhar [18], they utilized the FE method to investigate how axial forces and BM, in addition to internal pressure, influence the burst pressure of corroded pipelines, demonstrating that compressive axial forces and closing BM (which induce compression in corroded regions) critically reduce the burst capacity. In two numerical studies conducted by Shuai et al. [19,20], they investigated the local buckling resistance and compressive buckling strain of corroded X80 steel pipes under BM using nonlinear FE models and revealed the limitations of existing strain based deep corrosion defect methods. Ma et al. [21] deduced and verified analytical ratcheting limits for pressurized straight pipes under combined internal pressure and reversed BM, demonstrating an improved accuracy over KTA/ASME and RCC-MR codes. Zhang et al. [22] numerically assessed fracture behavior in a clad pipeline containing a canoe-shaped external girth weld crack under a pure bending moment and combined BM–internal pressure. The proposed strain-based Failure Assessment Diagram (FAD) reveals that BS 7910 is more conservative, especially for deep cracks. Hou et al. [23] investigated local buckling failure and the compressive strain capacity of dented submarine pipelines under bending and hydrostatic loads and proposed a regression equation for predicting the buckling strain capacity:

(2) $$\varepsilon_{c}^{Critical}\left(\%\right)=11.598{\left(\frac{t}{D}\right)}^{1.313}{\left[1-1.3\frac{\left(P_{e}-P_{i}\right)D}{2\sigma_{y}t}\right]}^{2}\left[12.528-17.832{\left(\frac{d}{D}\right)}^{0.343}\right]+0.293$$

where $\varepsilon_{c}^{Critical}$, P_e_, P_i_, and d denote the buckling strain capacity, external pressure (MPa), internal pressure [MPa], and dent depth [mm], respectively. In their another study [24], the ultimate BM of subsea pipelines with corrosion–dent coupled defects was assessed through analytical and numerical methods, and a high-order approximation method and plane strain-based analytical solution was proposed. Wang et al. [25] experimentally and numerically analyzed the buckling behavior and limit strain capacity of pipelines with kinked dents under BM, revealing that deep kinked dents significantly reduce the bending resistance and induce high compressive strains at the dent’s bottom during failure. Zhu et al. [26] utilized FE simulations considering damage mechanics to analyze how defect size (depth and circumferential length) affects the fracture performance of X80 pipeline girth welds under combined internal pressure and BM. Results indicated that defect depth had a more pronounced detrimental effect on failure load than circumferential length, with a sudden drop in failure load observed when the defect depth exceeded a critical threshold. Wang et al. [27] employed numerical modeling validated by field trials to analyze the BM of polyethylene (PE) pipelines subjected to traffic loading. An empirical method was proposed and validated via the literature case history to predict traffic-induced bending moments. Sun et al. [28] addressed the lack of failure pressure models for pipelines under the combined action of internal pressure, BM, and axial forces by proposing a unified yield criterion-based equation to assess the pipe’s failure pressure, integrating common operating loads through a three-dimensional stress model.

From the literature review, we have learned that many studies have focused on the buckling resistance of defected pipelines, particularly corroded pipelines. However, there is limited discussion on pipelines with dents. Additionally, the buckling resistance analysis of pipelines primarily relies on experimental and numerical simulation methods, which offer valuable insights into buckling behavior analysis. Nevertheless, due to the involvement of numerous variables in predicting the critical buckling moment (CBM) of dented pipelines, experimental and numerical approaches require intensive, case-specific simulations for each dent profile, load scenario, or material variation. The substantial resource and computational demands limit their applicability [29]. Recent artificial intelligence (AI) and ML breakthroughs offer innovative tools to meet these challenges. The data-driven models can not only overcome challenges posed by complex interactions among varied dent profiles and pipeline parameters but also leverage high-fidelity data from FE analyses or experiments to generalize predictions across diverse material behaviors and boundary conditions, eliminating the need for repetitive simulations tailored to each specific scenario. To the best of the authors’ knowledge, no research has discussed the CBM of dented pipelines under the combined action of internal pressure and BM, nor have they employed ML techniques to predict the CBM of dented pipelines.

This paper employs a novel FE-ML-based approach to investigate and predict the buckling behavior of dented pipelines under the actions of internal pressure and BM. A robust three-dimensional nonlinear FE model, incorporating an indenter and a pipeline, was established to analyze the CBM in an API 5L X52 steel dented pipeline. The developed FE model was then utilized to investigate the parametric effects of internal pressure, pipeline outer diameter and wall thickness, dent depth, and dent size on the CBM at the dent defect and to construct a dataset of CBM for the pipeline. Finally, a hybrid algorithm combining the Bald Eagle Search (BES), Lévy flight, and Simulated Annealing (SA) was implemented to enhance Extreme Learning Machine (ELM) optimization, achieving precise CBM prediction through synergistic algorithmic integration. The novelty of this study lies in the development of a hybrid numerical simulation and ML methodology, providing a comprehensive evaluation and prediction of the buckling behavior of dented pipelines subjected to complex loads.

The remaining sections are organized as follows: Section 2 develops the FE model and constructs the corresponding results dataset. Section 3 examines FE outcomes and their implications. Section 4 details the proposed ML methodology alongside its findings. Finally, Section 5 concludes with final remarks and key insights.

## 2. FE Modeling

The FE modeling in this section incorporates the following assumptions:

- The FE analysis only accounts for stable internal pressure and bending moment loads, excluding the effect of temperature variations and cyclic loads.
- The pipeline is modeled as an intact, defect-free structure. Weld seams and other connecting structures are ignored.
- The pipe material is considered as isotropic, homogeneous, and rate-independent.

### 2.1. General

**Figure 2:**
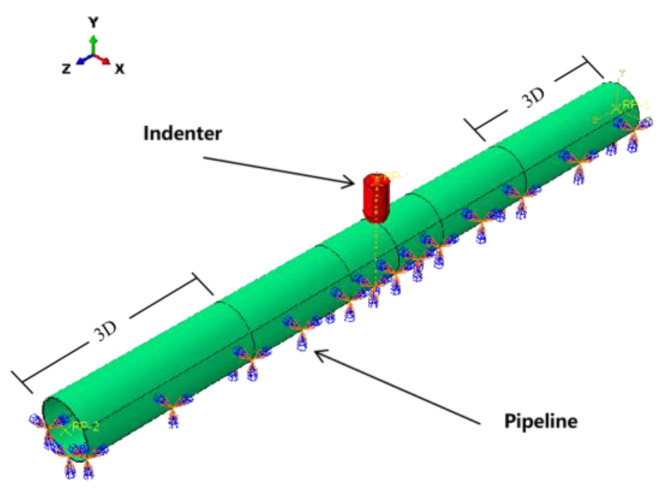
Illustration of a dented pipeline model.

A three-dimensional FE model of a dented pipeline, consisting of an indenter and a pipeline, was developed using ABAQUS software, as shown in Figure 2, where the X-Y plane and Z-direction are the pipeline’s cross-section and longitudinal direction, respectively. The indenter was represented by a rigid, spherical part, while the pipeline model’s length was calibrated to 12 times its diameter to eliminate end-structure effects on the outcomes.

**Figure 3:**
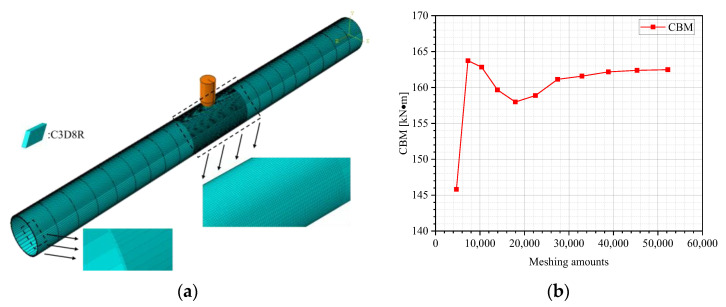
Meshing and its sensitivity analysis of the FE model: (**a**) model meshing; (**b**) meshing sensitivity analysis.

The indenter model was established using an analytical, rigid-type part, eliminating the requirement for meshing. For a balance between computational cost and accuracy, the pipeline model was meshed on an eight-node linear brick, reduced integration, and hourglass control (C3D8R) solid element. The pipeline’s wall thickness was discretized into four layers. Targeted mesh refinement was applied to the indenter–pipeline contact zone (7.8 mm axial × 6.8 mm circumferential dimensions), contrasted with a coarser mesh allocation elsewhere in the pipeline model. To ensure good convergence accuracy of the FE results, a mesh sensitivity analysis was conducted. By gradually varying the axial and circumferential meshing density in the local regions, it was found that the change in the CBM became negligible when the total amounts of elements exceeded 27,456. Based on a comprehensive consideration of the stability and computational cost, the optimal amount of elements for the model was determined to be 27,456, corresponding to 27,456 integration points and 34,710 nodes. Figure 3 depicts the meshing and its sensitivity analysis of the FE model.

To model the pipeline–indenter interaction, a surface-to-surface contact method was employed, designating the indenter’s exterior area as the master surface and the pipeline’s outer surface as the slave surface. A friction coefficient of 0.3 governed the interaction, enforcing hard normal contact while constraining sliding during analysis. The bottom section of the pipeline model, spanning 120°, was fully restrained until the constraint was released under rotation loading. Reference points on both end sides were coupled to the outer 3D regions of the pipeline and defined as the rigid body.

**Figure 4:**
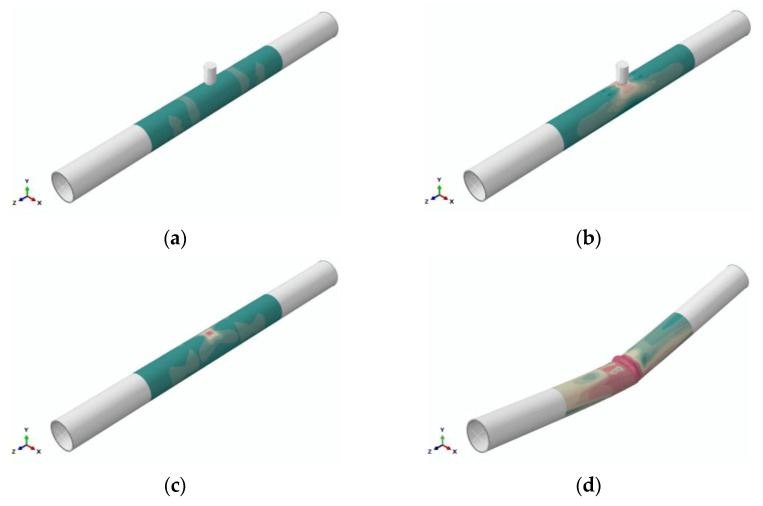
Analysis steps: (**a**) applying internal pressure; (**b**) contact and loading; (**c**) removing the indenter; and (**d**) bending.

A nonlinear quasi-static procedure, with a convergence tolerance of 10^−5^, was employed to conduct numerical simulations on the formation of unconstrained dents on pipelines and the buckling failure of dented pipelines. In practical engineering scenarios, unconstrained dents may occur when pipelines in service are impacted by falling rocks or excavation equipment. Hence, four general static steps were set up to analyze the buckling process of unconstrained dented pipelines, as illustrated in Figure 4.

Step 1: To model an in-service intact pipeline, a pressure load was applied to the pipeline’s inner surface.

Step 2: A displacement load via the indenter was administered in the negative Y-direction to fabricate an unconstrained dent on an operating pipeline.

Step 3: A displacement load in the positive Y-direction was employed to withdraw the indenter, completing the dent simulation.

Step 4: Rotation loads were assigned to the ends of the pipeline until buckling occurred.

### 2.2. Pipeline Material and Buckling Failure Criterion

For this study, API 5L X52 pipeline steel, which is widely used in global oil and gas transportation, was selected as the pipe material for the FE simulation. The stress–strain data of this steel was determined using the Ramberg–Osgood (R-O) equation [14]:

(3) $$\varepsilon =\frac{\sigma }{E}+\alpha \frac{\sigma }{E}{\left(\frac{\sigma }{\sigma_{y}}\right)}^{n-1}$$

where ε denotes strain, σ indicates stress [MPa], E is the elastic modulus of the pipe material [GPa], α represents the yield offset, and n is the strain-hardening exponent. The material properties of X52 pipeline steels are summarized in Table 1, and these parameters have been widely adopted in similar pipeline structural analyses.

**Figure 5:**
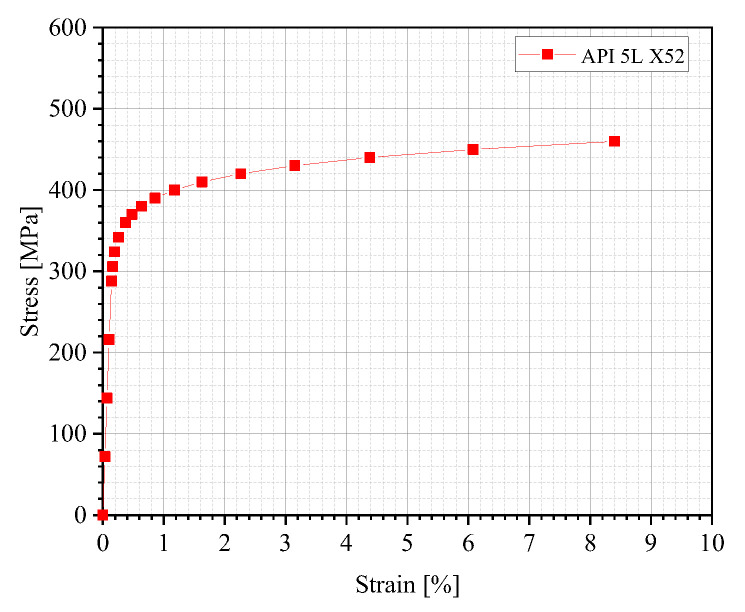
The true stress–strain curve.

The R-O equation yields the engineering stress–strain data of the material, which should be converted into the true stress–strain data by Equations (4) and (5) [14]. The true stress–strain curves are shown in Figure 5:

(4) $$\sigma_{true}=\sigma_{eng}\left(1+\varepsilon_{eng}\right)$$

(5) $$\varepsilon_{true}=ln\left(1+\varepsilon_{eng}\right)$$

where σ_true_, σ_eng_, ε_true_, and ε_eng_ denote the true stress, engineering stress, true strain, and engineering strain, respectively.

**Figure 6:**
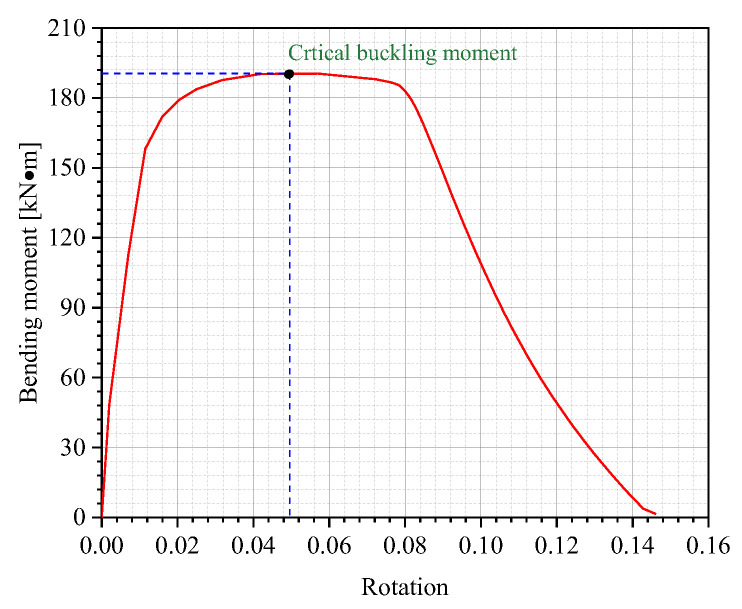
The moment–rotation curve.

When subjected to monotonically increasing bending moments, local buckling may occur either before or after the local pipe section reaches its full plastic resistance capacity. Therefore, under the combined action of internal pressure and bending loads, the compressed side of the pipeline becomes more susceptible to premature failure through buckling [31]. The moment–rotation curve serves as the most effective method for evaluating buckling behavior, as shown in Figure 6, where the maximum moment is defined as the critical buckling moment (CBM) in this study.

### 2.3. Parameter Setting

**Figure 7:**
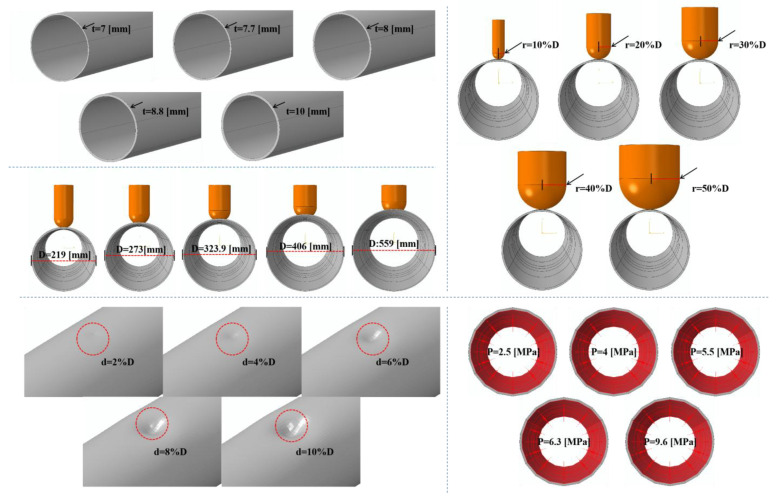
Parameter setting in the FE simulation.

Single-factor analysis approach-based parametric studies were implemented to examine the effect of the operating pressure (P), the pipe’s outer diameter (D) and wall thickness (t), indenter radius (r), and dent depth (d) on the CBM of dented pipelines. As shown in Figure 7, a dome indenter with radii equal to 10%, 20%, 30%, 40%, and 50% of the pipe diameter were employed to fabricate the dents with initial depths of 2%, 4%, 6%, 8%, and 10% of the pipe diameter, respectively; the FE analysis incorporated the actual dimensions of pipelines, with the outer diameters selected as 219 mm, 273 mm, 323.9 mm, 406 mm, and 559 mm and the wall thicknesses chosen as 5.6 mm, 7 mm, 8 mm, 8.8 mm, and 10 mm; and all dents were introduced in pipelines operating under non-cyclic pressures of 2.5 MPa, 4 MPa, 5.5 MPa, 6.3 MPa, and 9.6 MPa. Table 2 shows the base conditions of the parameters used in this study.

## 3. FE Results and Discussion

### 3.1. Model Validation

#### 3.1.1. Verification of Pipeline Dent Formation Process

**Figure 8:**
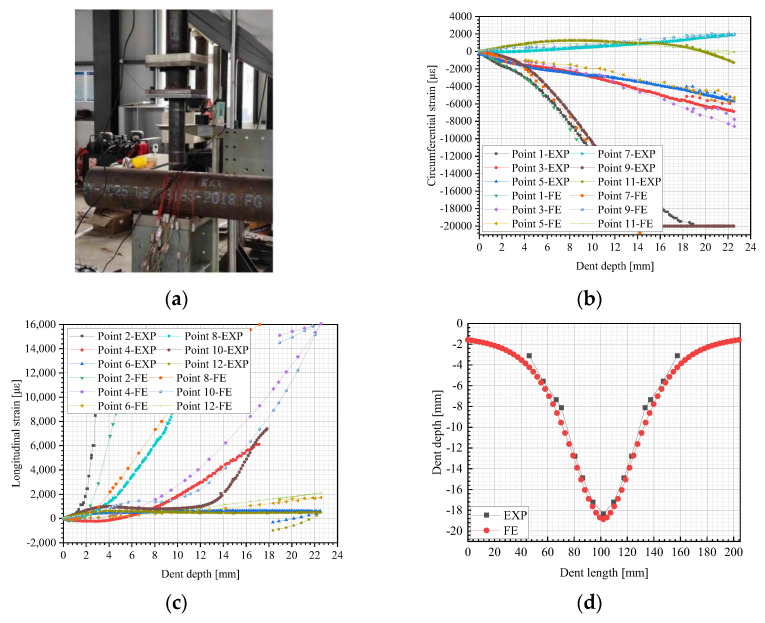
Comparison between the experimental data and FE results: (**a**) view of indentation test; (**b**) circumferential strain; (**c**) longitudinal strain; and (**d**) dent profile.

To assess the reliability of the FE model, an indentation test on a pipe with a length of 1000 mm and specifications of Φ219 mm × 6 mm was conducted, as illustrated in Figure 8a. The pipe material was Q235b steel, and a spherical indenter with a radius of 42.5 mm was used, creating an initial dent depth of 22 mm. Numerical calculations were performed, and the FE results were compared with the experimental data. The displacement–load curves during the indentation process, as well as the axial and circumferential strains of the pipe wall at the measurement positions, along with the corresponding FE model predictions, were recorded and compared, as shown in Figure 8. Figure 8b,c revealed that the numerical predictions were in good agreement with the experimental data. Furthermore, Figure 8d illustrated that the indentation test recorded a dent depth of 18.34 mm after the indenter removal, whereas the numerical simulation calculated 18.86 mm—yielding only a 2.8% difference between the experimental and numerical results. Therefore, the developed FE model can be deemed reasonable and be implemented for simulating the formation process of pipeline indentations.

#### 3.1.2. Verification of Pipeline Buckling Process

**Figure 9:**
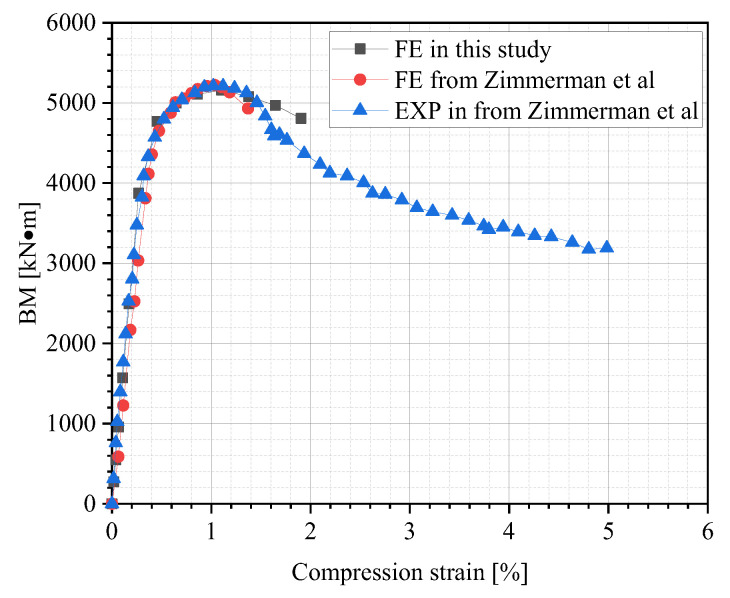
Comparison between the test data [32] and FE outcomes.

In a subsequent validation study, numerical simulations were conducted to replicate Zimmerman et al.’s [32] prior buckling experiments on an X80 steel pipe. The test utilized a pipe specimen with a length of 3200 mm, a diameter of 762 mm, and a wall thickness of 15.7 mm. This specimen was tested under conditions devoid of internal pressure or additional pre-loading. After numerical calculations, the FE results were compared with the experimental data. The comparison of strain–BM curves is shown in Figure 9. Figure 9 demonstrated that the test data were in good agreement with FE outcomes. Table 3 presents the relative error (RE), expressed in Equation (6), between the maximum BM results of the experiment and the simulation, which is 0.73%. This close correlation confirms the FE model’s reliability and establishes its suitability for simulating the pipeline buckling behavior:

(6) $$RE=\left(\frac{\left|{BM}_{test}-{BM}_{FE}\right|}{{BM}_{test}}\right)\times 100\%$$

where BM_test_ and BM_FE_ indicate the pipe’s maximum bending moment value from the test and numerical simulation, respectively.

### 3.2. Effects of Operating Pressure on CBM

**Figure 10:**
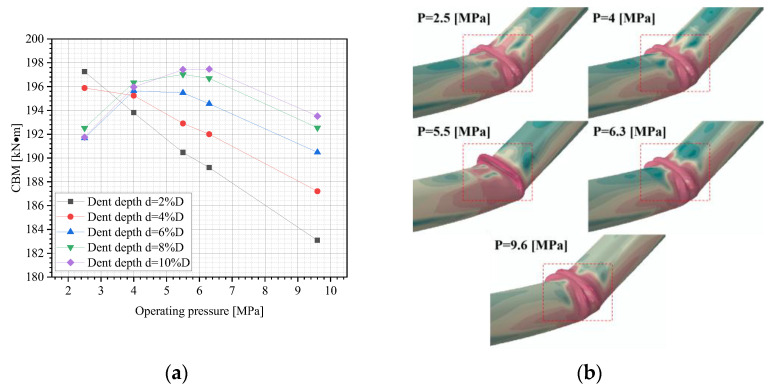
FE outcomes: (**a**) variations in the CBM of dented pipelines under different dent depths and internal pressures; (**b**) buckling modes of dented pipelines under various internal pressures (d = 2%D).

Figure 10a illustrates the variation in the CBM of dented pipelines with various dent depths and internal pressures. As is clear, when the dent is shallow (d = 2%D and 4%D), the CBM shows a monotonic decrease as the internal pressure increases. It might be because the lower internal pressure reduces the rebound magnitude of the dent after removing the indenter, resulting in a deeper inward bend at the dented region. This induces a certain degree of bending in the pipe body, thereby increasing the CBM. For deeper dents (d > 4%D), the CBM’s trend changes from an initial increase followed by a decrease as the internal pressure rises, as previously found by Shuai et al. [31]. This is attributed to the synergistic effects of pipeline stiffness and hoop stress on the pipe’s buckling resistance. At a lower internal pressure, pipeline stiffness is reduced, leading to poorer buckling resistance. As internal pressure increases, both pipeline stiffness and CBM rise. Beyond a threshold internal pressure, however, the hoop stress increases dominantly with further pressure elevation. This reduces the CBM because the critical load for cylindrical shells is proportional to the tangent modulus of the material at buckling. Higher hoop stress lowers the tangent modulus of the pipeline steel [33], ultimately diminishing the CBM.

The buckling modes of dented pipelines under different internal pressures are shown in Figure 10b. It can be seen that the buckling mode of the dented pipe varies under different internal pressures. It is because the presence of dents causes local ovality on the pipe’s cross-section. Various internal pressures lead to changes in the depth of the dents after rebound, resulting in different ovalization levels of the pipe, which affects the buckling mode of the pipe.

### 3.3. Effects of the Pipe’s Outer Diameter on CBM

**Figure 11:**
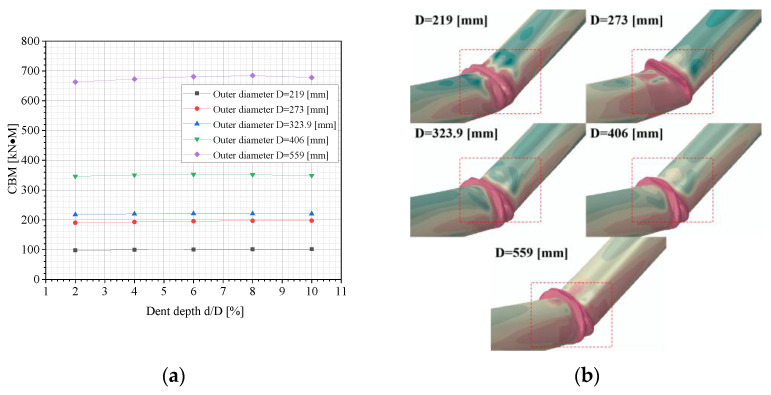
FE outcomes: (**a**) variations in the CBM of dented pipelines under different dent depths and pipe’s outer diameters; (**b**) buckling modes of dented pipelines under various pipes’ outer diameters (d = 2%D).

Figure 11a demonstrates the variation in the CBM of dented pipelines with various dent depths and the pipe’s outer diameters. In Figure 11a, an increase in dent depth has minimal impact on the CBM under identical pipe diameters, with only slight variations observed. As the pipe diameter increases, the CBM rises significantly. Particularly, when the pipe’s outer diameter reaches 559 mm, the CBM increases from an average of 350 kN·m at 406 mm to approximately 650 kN·m on average. This indicates that larger diameters enhance the pipe’s buckling resistance. The increase in the pipe’s outer diameter not only improves the pipe’s stiffness, strengthening its buckling resistance, but also affects the hoop-stress level under a given internal pressure. While the increased hoop stress might generally reduce buckling resistance, the impact of the pipe diameter in this context is more significant.

Figure 11b shows the local buckling mode of dented pipes under different pipe diameters. It is observed that the local buckling modes vary with the pipe diameter at identical dent depths: at a diameter of 219 mm, the local buckling mode manifests as “single inward”, while when the diameter increases to 273 mm, this changes to “single outward bulge”. With further diameter increases, the buckling mode evolves into “single inward with double outward bulges”. This demonstrates that the pipe diameter significantly influences the characteristics of local buckling deformation.

### 3.4. Effects of the Pipe’s Wall Thickness on CBM

**Figure 12:**
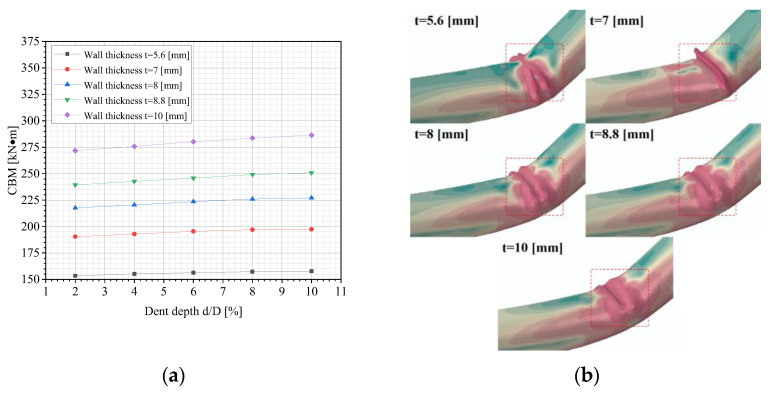
FE outcomes: (**a**) variations in the CBM of dented pipelines under different dent depths and pipe’s wall thicknesses; (**b**) buckling modes of dented pipelines under various pipes’ wall thicknesses (d = 2%D).

Figure 12a demonstrates the variation in the CBM of dented pipelines with various dent depths and the pipes’ wall thicknesses. It can be seen that thicker pipe walls are positively correlated with a higher CBM. Increased wall thickness enlarges the pipe’s cross-section, directly boosting the sectional moment of inertia, which is a key parameter for quantifying bending resistance. According to buckling theory, the CBM is proportional to the moment of inertia. Thus, thicker walls impede the occurrence of buckling. Additionally, thicker walls distribute the external load across more pipe material, mitigating the local stress concentration and allowing the pipeline to withstand higher bending moments without instability. The buckling modes of dented pipelines under different wall thicknesses of the pipe are illustrated in Figure 12b. It is evident that the buckling mode of the pipeline varies with different wall thicknesses.

### 3.5. Effects of Indenter Radius on CBM

**Figure 13:**
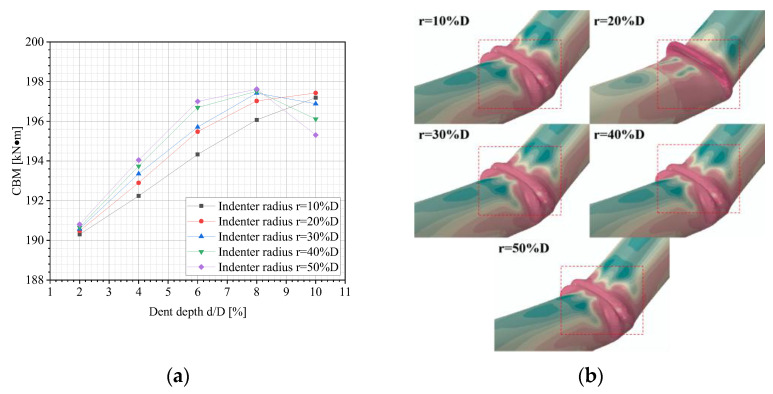
FE outcomes: (**a**) variations in the CBM of dented pipelines under different dent depths and indenter radii; (**b**) buckling modes of dented pipelines under various indenter radii (d = 2%D).

Figure 13a shows the variation in the CBM of dented pipelines with various dent depths and indenter radii. When the dent depth is shallow (d ≤ 2%D), the CBM under different indenter radii are close with minor differences. As the dent depth increases, all curves exhibit a rapidly increasing trend. This is because, at smaller dent depths, the structural integrity of the pipeline remains unaffected, and buckling behavior is primarily governed by the inherent strength of pipeline materials. Thus, the influence of the indenter radius is not yet evident. When the indenter radius exceeds 30%D, the curves show significant fluctuations. The CBM no longer changes monotonically with the dent depth but instead increases initially and then decreases, concluding that the indenter size has a minimal impact on the CBM of dented pipelines. The buckling modes of dented pipelines under different indenter radii are shown in Figure 13b. It is seen that the buckling mode of the pipeline is similar to that in Figure 12b.

## 4. ML Approach

**Figure 14:**
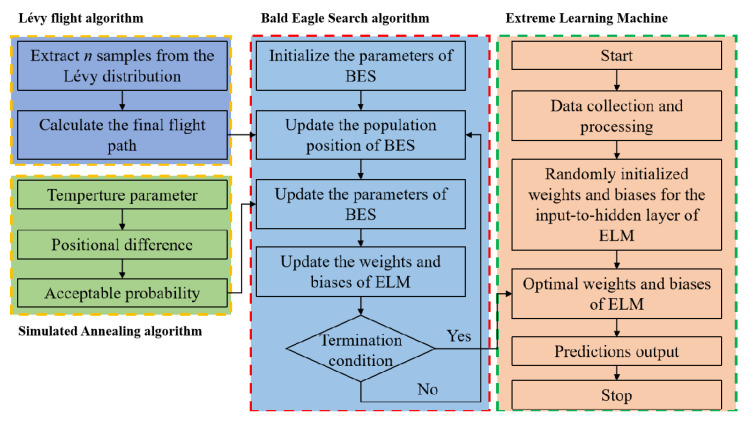
Flowchart of the LSBES-ELM model.

In this section, a novel Extreme Learning Machine (LSBES-ELM) model, optimized via three intelligent algorithms (Bald Eagle Search, Lévy flight, and Simulated Annealing), is proposed for the high-precision prediction of the pipe’s CBM. The proposed ML model comprises three steps: (1) data collection and processing, (2) development of the ML model, and (3) prediction results and performance evaluation of the ML model. Figure 14 illustrates the flowchart of the developed LSBES-ELM model.

### 4.1. Data Collection and Processing

Based on the numerical results in Section 3, a pipe’s CBM dataset is constructed. This dataset consists of six parameters: operating pressure (P), the pipe’s outer diameter (D) and wall thickness (t), dent depth (d), indenter radius (r), and CBM. The first five parameters (D, t, d, r, and P) serve as input variables, while the latter acts as the output variable. The ranges of input variables are detailed in Section 2.3. The dataset is randomly partitioned into training and test sets, with 80% allocated for training and 20% for testing. Furthermore, all data are normalized using the following equation [34]:

(7) $$x^{\prime }=\frac{\left(y_{\max}-y_{\min}\right)*\left(x-x_{\min}\right)}{\left(x_{\max}-x_{\min}\right)}+y_{\min}$$

where x′ is the normalized value of variable x; x_max_ and x_min_ denote the maximum and minimum values of variable x, respectively; and y_max_ and y_min_ represent the upper and lower limits of the normalization threshold, where 0 and 1 are taken, respectively.

### 4.2. ML Model Development

**Figure 15:**
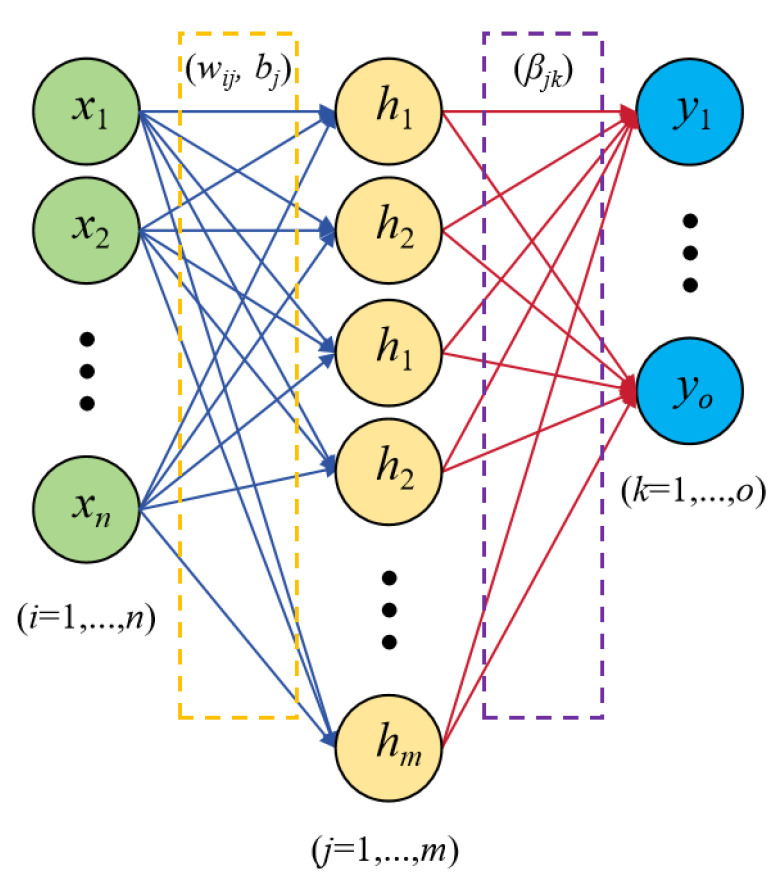
ELM model structure.

The Extreme Learning Machine [35] is employed as the benchmark model in this study. As a rapid learning algorithm tailored for single-hidden layer feedforward neural networks (SLFNs), the ELM model distinguishes itself from conventional feedforward neural networks (FFNNs) through its unique structural design [34,35], as shown in Figure 15.

Specifically, the ELM framework employs deterministic mappings for hidden-output layer connections, while utilizing randomized structural configurations in other components to optimize computational efficiency. A unique characteristic of the ELM model is its elimination of backpropagation (BP)-based weight and bias training. Instead, the model employs randomly initialized parameters (weights and biases) for the input-to-hidden layer, followed by the determination of the hidden-to-output layer’s weights through least squares minimization between the predicted and true values [36]. This enables ELM to achieve training speeds exceeding traditional BP neural networks by an order of magnitude. The mathematical equations governing the ELM model are expressed as follows [34,35]:

(8) $$H\left(x\right)=\left(h_{1}\left(x\right),\ldots ,h_{m}\left(x\right)\right)$$

(9) $$h_{i}\left(x\right)=g\left(w_{i},\;b_{i},\;x\right)=g\left(w_{i}x+b_{i}\right)$$

(10) $$Y=\sum_{i=1}^{m}\beta_{i}h_{i}\left(x\right)=H\left(x\right)\beta ,\;\beta =\left[\begin{array}{cccc} \beta_{1} & \beta_{2} & \ldots & \beta_{m} \end{array}\right]$$

(11) $$H=\left[\begin{array}{cccc} g\left(w_{1}x_{1}+b_{1}\right) & g\left(w_{2}x_{1}+b_{2}\right) & \ldots & g\left(w_{m}x_{1}+b_{m}\right)\\ g\left(w_{1}x_{2}+b_{1}\right) & g\left(w_{2}x_{2}+b_{2}\right) & \ldots & g\left(w_{m}x_{2}+b_{m}\right)\\ \vdots & \vdots & \ddots & \vdots \\ g\left(w_{1}x_{n}+b_{1}\right) & g\left(w_{2}x_{n}+b_{2}\right) & \ldots & g\left(w_{m}x_{n}+b_{m}\right) \end{array}\right]$$

where H(x), h_m_(x), g, β, w, b, and Y denote the hidden layer output, the output of the mth neuron in the hidden layer, the activation function, the weight of the hidden-to-output layer, the weight of the input-to-hidden layer, the hidden layer bias, and the expected output of the output layer, respectively.

**Figure 16:**
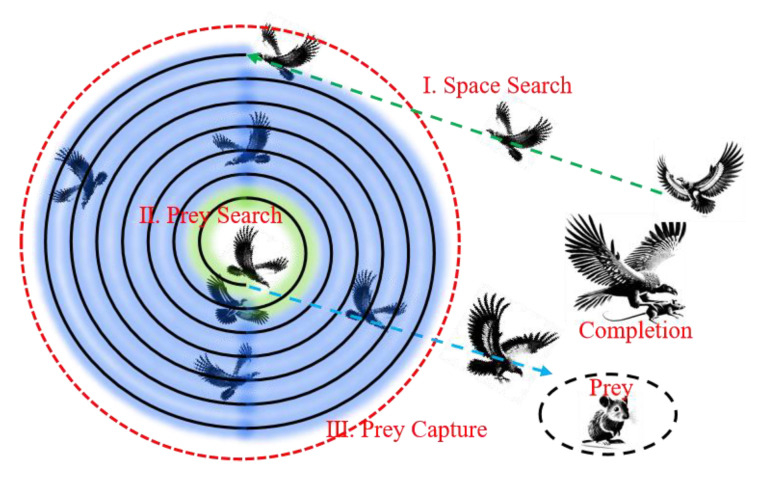
Flowchart of BES algorithm.

However, the random initialization of weights and biases may introduce output instability in the ELM model. To mitigate this, a Bald Eagle Search (BES) [37] intelligent algorithm is employed to optimize the hidden-layer parameters through continuous position updates, enhancing the stability and performance of the ELM model. The BES algorithm was proposed by Alsattar et al. [38], inspired by the natural hunting behavior of bald eagles, and is divided into three sequential phases: I. Space Searching; II. Prey Searching; and III. Diving Capture, as illustrated in Figure 16.

In the Space Searching phase, the initial parameter exploration is established using the position-updating mechanism, which is mathematically expressed as follows [38]:

(12) $$P_{i,\mathrm{new}}=P_{\mathrm{best}}+\alpha \rho \left(P_{\mathrm{mean}}-P_{i}\right)$$

where P_i, new_ and P_i_ are the latest and current positions of the bald eagle swarm; P_best_ signifies the optimal positional coordinate attained by the bald eagle during iteration; α denotes a position-adaptive control coefficient with the range of (1.5, 2); ρ represents a stochastic parameter uniformly sampled from the interval (0, 1); and P_mean_ corresponds to the centroid of the bald eagle swarm’s positional distribution.

In the Prey Searching phase, the BES algorithm leverages a spiral contraction mechanism to progressively reduce the search domain from the outer region toward the center, pinpointing the optimal dive initiation point for prey capture—significantly minimizing redundancy in the search process. This phase is governed by the following equations [38]:

(13) $$x\left(i\right)=r\left(i\right)\sin\left(\theta \left(i\right)\right)=\left(\theta \left(i\right)+R\rho \right)\frac{\sin\left(a\pi \rho \right)}{max\left(\left|x\rho \right|\right)}$$

(14) $$y\left(i\right)=r\left(i\right)\cos\left(\theta \left(i\right)\right)=\left(\theta \left(i\right)+R\rho \right)\cos\frac{\left(a\pi \rho \right)}{max\left(\left|y\rho \right|\right)}$$

(15) $$P_{i,\mathrm{new}}=P_{i}+x\left(i\right)\left(P_{i}-P_{\mathrm{mean}}\right)+y\left(i\right)\cdot \left(P_{i}{-P}_{\mathrm{best}}\right)$$

where x(i) and y(i) represent the position of the bald eagle in polar coordinates, respectively; θ(i) and r(i) indicate the polar angle and polar diameter of the spiral equation, respectively; and a and R are flight trajectory parameters. However, the BES algorithm faces challenges, including a restricted search space during its initial phase and subpar precision in local search during the second phase. To address these issues, we introduce the Lévy flight algorithm [39]. The Lévy flight algorithm originates from Lévy’s symmetric stable distribution integral, which is a special method for generating random step sizes, as expressed below [39]:

(16) $$Lé\mathrm{vy}\left(s\right)\sim u=t^{-1-\varphi }$$

(17) $$x_{i+1}=x_{i}+\lambda \cdot \frac{u}{|v{|}^{\frac{1}{\varphi }}}$$

(18) $$P_{i,\mathrm{new}}=P_{\mathrm{best}}+\alpha \rho \left(P_{\max}-P_{i}\right)\times Lé\mathrm{vy}$$

where u and φ donate the scale parameter and shape parameter of the Lévy distribution, respectively, and v represents a random variable following a standard normal distribution. The dual strategy mechanism of the Lévy flight algorithm combined with long-distance exploration can expand the search space of the BES algorithm and improve its local search accuracy.

In the Diving Capture phase, the BES algorithm models the bald eagle’s swift descent to directly determine the optimal parameter configuration [38]:

(19) $$x_{1}\left(i\right)=r\left(i\right)\sin\left(h\left(\theta \left(i\right)\right)\right)/\max\left(\left|x\rho \right|\right)$$

(20) $$y_{1}\left(i\right)=r\left(i\right)\cos\left(h\left(\theta \left(i\right)\right)\right)/\max\left(\left|x\rho \right|\right)$$

(21) $$\delta_{x}=x_{1}\left(i\right)\left(P_{i}{-c}_{1}P_{\mathrm{mean}}\right),\;\delta_{y}=y_{1}\left(i\right)\left(P_{i}-c_{2}P_{\mathrm{best}}\right)$$

(22) $$P_{i,\mathrm{new}}=\rho P_{\mathrm{best}}+\delta_{x}+\delta_{y}$$

where δ_x_ and δ_y_ represent the positional offsets of the bald eagle relative to its current location, respectively, and c_1_ is the intensity of motion towards the optimal position, while c_2_ denotes the intensity of motion towards the center. To avoid local optima, a Simulated Annealing (SA) algorithm based on specific acceptance probabilities is introduced [40]. The algorithm can prevent the BES algorithm from being limited to local regions [35,40]:

(23) $$p=\exp\left(\frac{E_{\mathrm{new}}-E_{\mathrm{old}}}{T}\right)$$

where p donates the probability of accepting a worse solution; E_new_ represents the energy of the new solution, while E_old_ is the energies of the old solution; and T is the current temperature.

In this study, the LSBES-ELM model comprises three layers: an input layer with 5 neurons, a hidden layer containing 30 neurons, and an output layer with 1 neuron. The sigmoid function serves as the activation function. For hyperparameter configuration, the LSBES-ELM model employs the population size (40), maximum iterations (30), Lévy flight parameter (2), position update coefficient (10), rotation radius adjustment parameter (1.5), tolerance threshold (1 × 10^−4^), and maximum temperature (5).

To evaluate the predictive performance of the LSBES-ELM model, six error metrics [34,37], including the Mean Relative Error (MRE), Mean Absolute Percentage Error (MAPE), Mean Square Error (MSE), Root Mean Square Error (RMSE), Mean Absolute Error (MAE), and coefficient of determination (R^2^), were adopted to measure the deviation between the model’s predicted results and the true values:

(24) $$\mathrm{MRE}=\frac{1}{n}\sum_{i=1}^{n}\frac{\left|y_{i}-y_{i}^{\prime }\right|}{y_{i}}\times 100\%$$

(25) $$\mathrm{MAPE}=\frac{100\%}{n}\sum_{i=1}^{n}\left|\frac{y_{i}-y_{i}^{\prime }}{y_{i}}\right|$$

(26) $$\mathrm{MSE}=\frac{1}{n}\sum_{i=1}^{n}{\left(y_{i}-y_{i}^{\prime }\right)}^{2}$$

(27) $$\mathrm{RMSE}=\sqrt{\frac{1}{n}\sum_{i=1}^{n}{\left(y_{i}-y_{i}^{\prime }\right)}^{2}}$$

(28) $$\mathrm{MAE}=\frac{1}{n}\sum_{i=1}^{n}\left|y_{i}-y_{i}^{\prime }\right|$$

(29) $$R^{2}=1-\frac{\sum_{i=1}^{n}{\left(y_{i}-y_{i}^{\prime }\right)}^{2}}{\sum_{i=1}^{n}{\left(y_{i}-\hat{y_{i}}\right)}^{2}}$$

where $y_{i}$, $y_{i}^{\prime }$, and $\hat{y_{i}}$ denote the prediction results, the true value, and the mean value, respectively; n represents the number of input variables. The results are presented in Section 4.

### 4.3. ML Predictions and Discussion

#### 4.3.1. Effects of Training Set Proportion on ML Predictions

**Figure 17:**
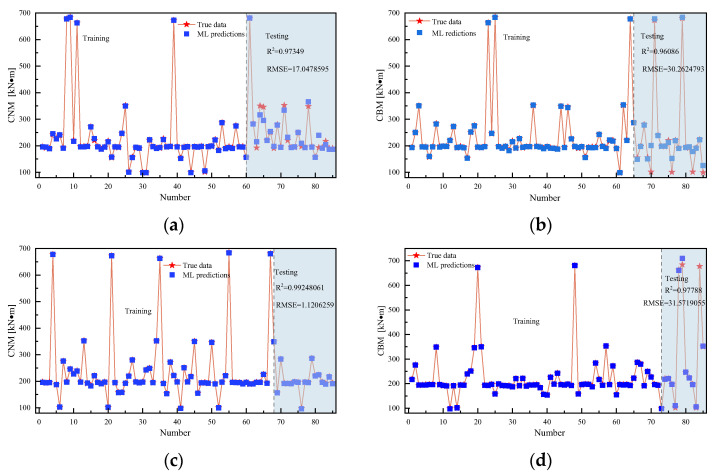
Training set proportion: (**a**) 70%; (**b**) 75%; (**c**) 80%; and (**d**) 85%.

Figure 17 illustrates how the LSBES-ELM model performs under different training set proportions. Figure 17 confirms the model’s consistent high accuracy and robust capacity to model nonlinear relationships in complex datasets. Crucially, the best performance occurs at 80% of the training set proportion (R^2^ = 0.992, RMSE = 1.121), indicating the excellent generalization ability in the presence of sufficient data, highlighting the key balance the model achieves between overfitting and underfitting.

#### 4.3.2. Effects of ELM’s Hidden Neurons and BES’s Population Size on ML Predictions

**Figure 18:**
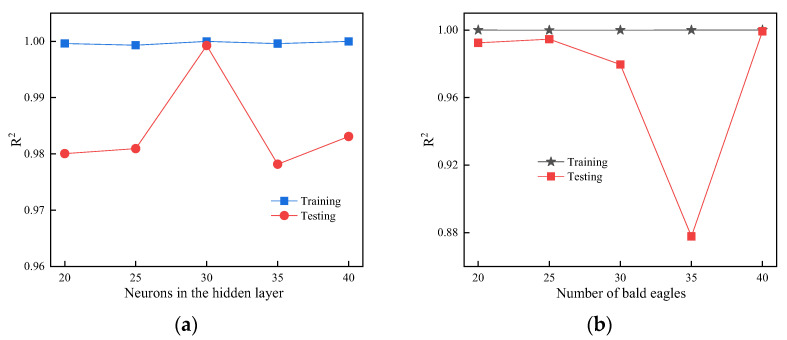
Parametric effects: (**a**) ELM hidden layer neurons; (**b**) BES population sizes.

The effects of ELM hidden layer neurons and BES population size on prediction results are shown in Figure 18. Figure 18a demonstrates that the optimal performance is achieved with 30 hidden layer neurons, while other configurations show little variation in R^2^ values, a trend consistent with our previous findings [35]. Figure 18b indicates that a population size of 40 bald eagles performs best, whereas with 35 bald eagles, a significant performance mismatch emerged between the test (R^2^ = 0.88) and training (R^2^ = 0.99) sets. The discrepancy stems from the algorithm’s entrapment in confined search regions, causing convergence to suboptimal minima rather than global solutions.

#### 4.3.3. Accuracy Evaluation of ML Predictions

**Figure 19:**
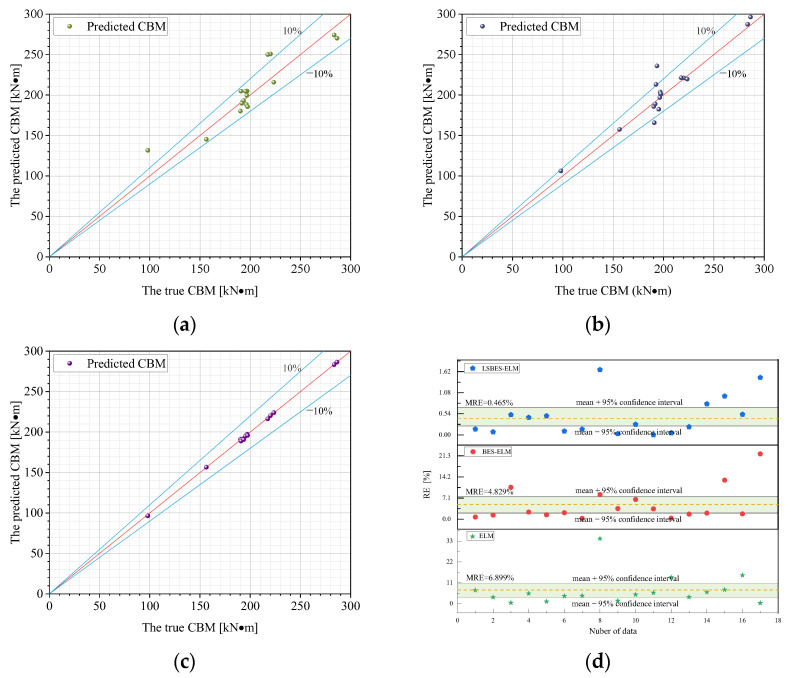
Comparison between the predicted results and true values: (**a**) ELM; (**b**) BES-ELM; (**c**) LSBES-ELM; and (**d**) RE.

Figure 19 presents the comparison of the pipe’s CBM predictions from the ELM, BES-ELM, and LSBES-ELM model against actual data. As observed in Figure 19a,b, the ELM and BES-ELM models exhibit a relatively low prediction accuracy, with some predicted values deviating more than ±10% from the true data. Figure 19c demonstrates that the LSBES-ELM model achieves a high degree of alignment between predicted and actual results, where nearly all data points cluster closely around the ideal fitting line. A tighter distribution near the y = x line indicates a superior prediction accuracy, as high-quality models tend to produce predictions scattered near this reference line. This means that the developed LSBES-ELM model possesses robust capabilities for accurate predictions on unfamiliar data. Figure 19d shows the RE of the ELM, BES-ELM, and LSBES-ELM model. As is clear, the ELM model exhibits the widest RE range [0.22, 34.25] with an MRE of 6.9%. The BES-ELM model shows an improved performance with an RE range of [0.28, 21.93] and an MRE of 4.83. Notably, the LSBES-ELM model achieves the smallest error margins, with an RE range of [0.0013, 1.47] and an MRE of 0.46%. This indicates that, while the BES algorithm enhances the ELM’s prediction accuracy, the integration of the Lévy flight and SA strategies further significantly boosts the performance of the BES algorithm. As a result, the LSBES-ELM model achieves minimal errors, demonstrating its effectiveness and reliability in predicting the CBM for dented pipelines.

#### 4.3.4. Performance Evaluation of ML Predictions

**Figure 20:**
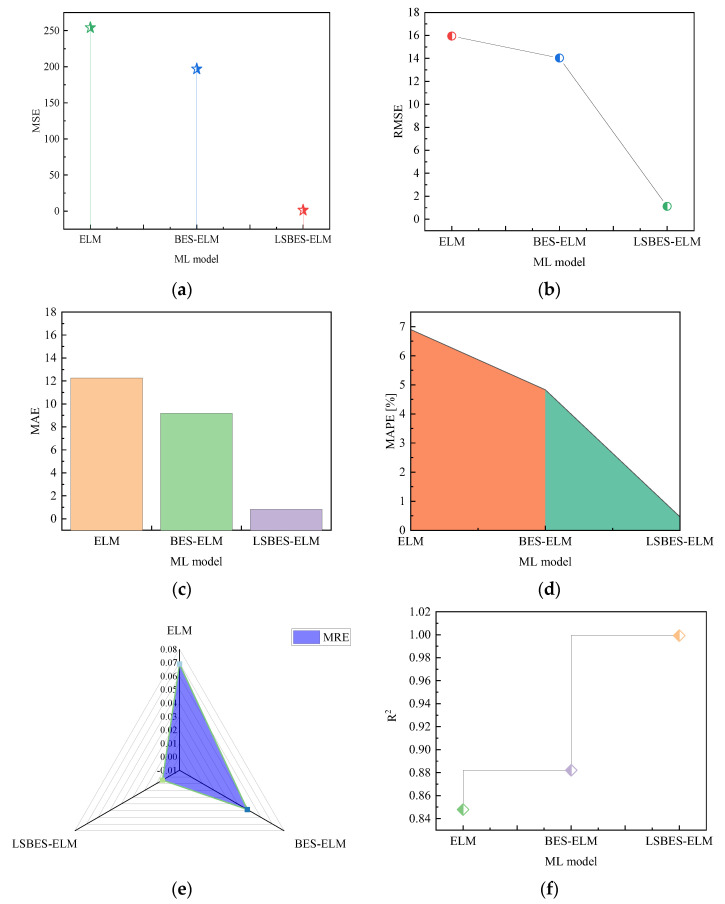
Error metrics: (**a**) MSE; (**b**) RMSE; (**c**) MAE; (**d**) MAPE; (**e**) MRE; and (**f**) R^2^.

Figure 18 illustrates the error metrics of the prediction results for the ELM, BES-ELM, and LSBES-ELM model. As shown in Figure 20a–e, the LSBES-ELM model demonstrates a superior performance across the MSE, RMSE, MAE, MAPE, and MRE. Specifically, its values for these metrics are 1.26, 1.12, 0.82, 0.46, and 0.0046, respectively, all of which are the lowest among all models. From Figure 20f, it is observed that the LSBES-ELM model achieves the highest R^2^ value of 0.992. Compared to the ELM and BES-ELM model, the LSBES-ELM model represents an improvement of 17.86% and 13.29%, respectively. The results indicate that the LSBES-ELM model exhibits the highest predictive performance and the smallest error margins for predicting the CBM in dented pipelines, outperforming both the ELM and BES-ELM model. The superiority of the LSBES-ELM model stems from its synergistic use of the Lévy flight and SA algorithm, which enhances the efficient traversal of the search space toward global optima. In contrast, the BES-ELM model relies solely on the BES algorithm, and the ELM lacks any supplementary optimization mechanisms. Consequently, the LSBES-ELM model achieves a superior predictive accuracy.

## 5. Conclusions

In this study, a novel FE-ML-based approach was developed to investigate and predict the buckling behavior of dented pipelines under the actions of internal pressure and BM. The parametric effects on the CBM of pipelines were quantified, and a CBM dataset was constructed. The integration of the BES, Lévy flight, and SA algorithm optimized the ELM model to achieve the accurate CBM prediction. The main conclusions of this study are drawn as follows:

(1) The CBM of dented pipelines exhibits a significant positive correlation with pipe diameter and wall thickness. This means that increasing the pipe diameter and wall thickness significantly enhances the pipes’ buckling resistance, enabling them to withstand higher bending moments before instability occurs. 

(2) The relationship between the internal pressure and CBM depends on the dent depth. For shallow dents, the CBM decreases monotonically as the internal pressure rises, while for deeper dents, the CBM changes from an initial increase followed by a decrease. This is attributed to the synergistic effect of pipe stiffness and circumferential stress on pipe’s buckling resistance. The indenter size has a minimal effect on the CBM of dented pipelines.

(3) The LSBES-ELM model achieves an R^2^ value of 0.992, surpassing the ELM and BES-ELM models by 17.86% and 13.29%, respectively. This marked enhancement in predictive performance results from synergistically integrating Lévy flight and SA strategies within the BES algorithm, substantially elevating the ELM’s accuracy.

(4) Compared with the ELM and BES-ELM model, the LSBES-ELM model has the lowest MSE of 1.26, RMSE of 1.12, MAE of 0.82, MAPE of 0.46, RE range of [0.0013, 1.47], and MRE of 0.0046, indicating its excellent performance in predicting the CBM of dented pipelines.

The findings of this study can be used to support the evaluation and decision-making of dented pipelines under buckling deformation scenarios. However, the limitation of this study is that the performance of the proposed ML model is constrained by the limited availability of training data, which inherently restricts its predictive accuracy and robustness. To advance the model’s reliability and precision, a critical priority lies in expanding the dataset through additional empirical or simulated data collection. This expansion is essential not only to validate the model’s consistency across diverse operational scenarios but also to rigorously assess its generalization capabilities, thereby ensuring its practical applicability in real-world engineering contexts where data variability and noise may present unforeseen challenges. The core contribution of this study is the proposal and in-depth analysis of the LSBES-ELM model, intentionally scoped to its unique structure and intrinsic performance; extensive comparisons are positioned as a key future direction.

## Figures and Tables

**Table 1:** Material properties of API 5L X52 pipeline steels [30].

Young’s Modulus E [GPa]	Poisson’s Ratio	Yield Offset ɑ	Strain-Hardening Exponent n	Yield Strength σ_y_ [MPa]	Ultimate Tensile Strength σ_u_ [MPa]
210	0.3	1.699	14.14	360	460

**Table 2:** Base conditions of FE modeling in this study.

Variable	Operating Pressure [MPa]	Pipe Outer Diameter [mm]	Pipe Wall Thickness [mm]	Indenter Radius [mm]	Dent Depth [mm]
Value	5.5	273	7	54.6	5.46, 10.92, 16.38, 21.84, 27.3

**Table 3:** Specific results comparison.

Maximum BM [kN·m]				
EXP	FE in Ref. [32]	FE in This Study	RE in Ref. [32]	RE in This Study
5198	5247	5160	0.9%	0.73%

## Data Availability

The original contributions presented in this study are included in the article. Further inquiries can be directed to the corresponding authors.

## Nomenclature

The following nomenclatures are used in this manuscript:

FE	Finite element
ML	Machine learning
CNM	Critical buckling moment
BM	Bending moment
ELM	Extreme learning machine
BES	Bald eagle search
SA	Simulated annealing
ILI	Ln-line inspection
P_limit_	Burst pressure
σ_u_	Ultimate tensile strength
σ_y_	Yield strength
D	Pipe’s wall thickness
t	Pipe’s outer diameter
F	Axial force,
M	Bending moment
δ	Corrosion depth
W	Corrosion width
L	Corrosion length
FAD	Failure assessment diagram
$\varepsilon_{c}^{Critical}$	Buckling strain capacity
P_e_	External pressure
P_i_	Internal pressure
d	Dent depth
PE	Polyethylene
AL	Artificial intelligence
C3D8R	8-node linear brick, reduced integration, hourglass control
R-O	Ramberg-Osgood
ε	Strain
σ	Stress
E	Elastic modulus
α	Yield offset
n	Strain-hardening exponent
σ_true_	True stress
σ_eng_	Engineering stress
ε_true_	True strain
ε_eng_	Engineering strain
P	Operating pressure
r	Indenter radius
RE	Relative error
BM_test_	Pipe’s maximum bending moment from the test
BM_FE_	Pipe’s maximum bending moment from the numerical simulation
x	Variable
x′	Normalized value of variable x
x_max_	Maximum values of variable x
x_min_	Minimum values of variable x
y_max_	Upper limit of the normalization threshold
y_min_	Lower limit of the normalization threshold
SLFNs	Single hidden layer feedforward neural networks
FFNNs	Feedforward neural networks
BP	Backpropagation
H(x)	Hidden layer output
h_m_(x)	Output of the mth neuron in the hidden layer
g	Activation function
β	Weight of the hidden-to-output layer
w	Weight of the input-to-hidden layer
b	Hidden layer bias
Y	Expected output of the output layer
P_i, new_	Latest position of the bald eagle swarm
P_i_	Current position of the bald eagle swarm
P_best_	Optimal positional coordinate attained by the bald eagle during iteration
α	Position-adaptive control coefficient
ρ	Stochastic parameter
P_mean_	Centroid of the bald eagle swarm’s positional distribution
x(i)	Position of the bald eagle in polar coordinates
y(i)	Position of the bald eagle in polar coordinates
θ(i)	Polar angle of the spiral equation
r(i)	Polar diameter of the spiral equation
a	Flight trajectory parameter
R	Flight trajectory parameter
u	Scale parameter of the Lévy distribution
φ	Shape parameter of the Lévy distribution
v	Random variable
δ_x_	Positional offset of the bald eagle relative to its current location
δ_y_	Positional offset of the bald eagle relative to its current location
c_1_	Intensity of motion towards the optimal position
c_2_	Intensity of motion towards the center
p	Probability of accepting a worse solution
E_new_	Energy of the new solution
E_old_	Energy of the old solution
T	Current temperature
MRE	Mean relative error
MAPE	Mean absolute percentage error
MSE	Mean square error
RMSE	Root mean square error
MAE	Mean absolute error
R^2^	Coefficient of determination
y_i_	Prediction results
$y_{i}^{\prime }$	True value
$\hat{y_{i}}$	Mean value
n	Number of input variables
